# Antisense lncRNA LDLRAD4-AS1 promotes metastasis by decreasing the expression of LDLRAD4 and predicts a poor prognosis in colorectal cancer

**DOI:** 10.1038/s41419-020-2338-y

**Published:** 2020-02-28

**Authors:** Shaobo Mo, Long Zhang, Weixing Dai, Lingyu Han, Renjie Wang, Wenqiang Xiang, Zhimin Wang, Qingguo Li, Jun Yu, Jihang Yuan, Sanjun Cai, Guoxiang Cai

**Affiliations:** 1 Department of Colorectal Surgery, Fudan University Shanghai Cancer Center, Shanghai, 200032 China; 2 Department of Oncology, Shanghai Medical College, Fudan University, Shanghai, 200032 China; 3 Department of Cancer Institute, Fudan University Shanghai Cancer Center, Fudan University, Shanghai, 200032 China; 4 Shanghai-MOST Key Laboratory of Health and Disease Genomics, Chinese National Human Genome Center and Shanghai Industrial Technology Institute (SITI), Shanghai, 201203 China; 5 Department of Surgery, Johns Hopkins University School of Medicine, Baltimore, MD 21287 USA; 6 Department of Medical Genetics, Second Military Medical University, Shanghai, 200433 China

**Keywords:** Colorectal cancer, Long non-coding RNAs

## Abstract

Long noncoding RNAs (lncRNAs) have been revealed to play critical roles in tumor initiation and progression. The antisense lncRNA LDLRAD4-AS1 is the longest lncRNA of LDLRAD4, and its expression levels, cellular localization, precise function, and mechanism in colorectal cancer (CRC) remain unknown. In this study, we observed that lncRNA LDLRAD4-AS1 was located in the nucleus of CRC cells and that lncRNA LDLRAD4-AS1 was upregulated in most CRC specimens and cell lines. Overexpression of lncRNA LDLRAD4-AS1 was correlated with poor prognosis in CRC patients. LncRNA LDLRAD4-AS1 upregulation enhanced the migration and invasion of CRC cells in vitro and facilitated CRC metastasis in vivo. Mechanistic investigations suggested that lncRNA LDLRAD4-AS1 could decrease the expression of LDLRAD4 by disrupting the stability of LDLRAD4 mRNA, resulting in epithelial-to-mesenchymal transition (EMT) through upregulating Snail, thereby promoting metastasis in CRC. Our results demonstrated a previously unrecognized LDLRAD4-AS1-LDLRAD4-Snail regulatory axis involved in epigenetic and posttranscriptional regulation that contributes to CRC progression and metastasis.

## Introduction

Recognized as the third most commonly diagnosed malignant tumor, colorectal cancer (CRC) is the second leading cause of cancer-related death worldwide in both sexes^1^. According to Global Cancer Statistics 2018, over 1.8 million newly diagnosed CRC cases (10.2% for incidence) and 881,000 estimated deaths (9.2% in terms of mortality) were presumed to occur in 2018^2^. Due to the ineffectiveness of diagnostic methods and prognostic biomarkers and early relapse (local recurrence and distant metastasis), the prognosis of advanced-stage CRC patients remains far from satisfactory, despite great efforts in numerous clinical and basic studies. Tumor metastasis is a complicated process involving multiple genetic and epigenetic variations, leading to inferior biological outcomes^3^. However, the detailed molecular mechanisms implicated in the progression and metastasis of CRC are still obscure. Thus, a better understanding of the molecular mechanisms underlying CRC carcinogenesis and progression is essential for the development of CRC-specific diagnostic markers and effective therapeutic strategies for CRC patients.

Current research shows that only 2% of genomic transcripts have protein-coding ability; the remaining 98% of genomic transcripts do not have protein-coding function but play a crucial role in various biological processes regulating gene transcription, and they are called ncRNAs (noncoding RNAs)^4^. Defined by their length, long noncoding RNAs (lncRNAs) (200 nt–10 kb) account for >80% of ncRNAs^5–7^. Mounting evidence suggests that lncRNAs are involved in tumorigenesis^8,9^, tumor cell proliferation^10,11^, invasion^12^, migration^13^, apoptosis^14^, and angiogenesis^15^. Recent research has focused on the function of lncRNAs in human cancer pathogenesis^7,16,17^; antisense (AS) lncRNAs are reverse complements of their endogenous sense counterparts and comprise a substantial proportion of the entire long noncoding transcriptome^7^. Previous studies have revealed that antisense lncRNAs are involved in the tumorigenesis and development of cancer by regulating the expression of their endogenous sense genes. For instance, Zhang et al.^18^ found that lncRNA FOXC2-AS1 increased the expression of transcription factor FOXC2, further facilitating ABCB1 expression resulting in doxorubicin resistance in osteosarcoma. Yang et al.^19^ revealed that HOXD-AS1 suppressed HOXD3 transcription by recruiting PRC2 during CRC carcinogenesis and progression to induce the accumulation of the repressive marker H3K27me3 at the HOXD3 promoter. Although various lncRNAs have been discovered in recent decades, many lncRNAs and their functions or mechanisms remain unknown. The role of potential lncRNAs has stimulated intense scientific interest.

The LDLRAD4 (low density lipoprotein receptor class A domain containing 4) gene^20^, also known as C18orf1, was identified on chromosome 18p11.2 and contains eight potential antisense lncRNAs, of which the antisense lncRNA LDLRAD4-AS1 is the longest. However, whether lncRNA LDLRAD4-AS1 regulates the expression of LDLRAD4 and is related to prognosis in CRC remains unclear. In this study, we evaluated the pathophysiological function of lncRNA LDLRAD4-AS1 in CRC. We found that high lncRNA LDLRAD4-AS1 expression was associated with CRC progression and predicted poor prognosis. In addition, we demonstrated that high lncRNA LDLRAD4-AS1 expression promotes metastasis by decreasing the expression of LDLRAD4, resulting in epithelial-to-mesenchymal transition (EMT). LncRNA LDLRAD4-AS1 silencing inhibits CRC cell migration and invasion, while lncRNA LDLRAD4-AS1 overexpression promotes CRC cell migration and invasion. Our findings suggest that lncRNA LDLRAD4-AS1 could be a potential biomarker and treatment target in CRC.

## Results

### High expression of lncRNA LDLRAD4-AS1 is associated with the poor prognosis of CRC patients

In this study, we measured the expression level of 8 potential antisense lncRNAs in 20 paired CRC tumor tissues and adjacent nontumor colorectal tissues using qRT-PCR. These results indicated that lncRNA LDLRAD4-AS1 expression levels in CRC tumor tissues were significantly increased compared with those in adjacent nontumor colorectal tissues (Supplementary Fig. 1).

**Fig. 1:**
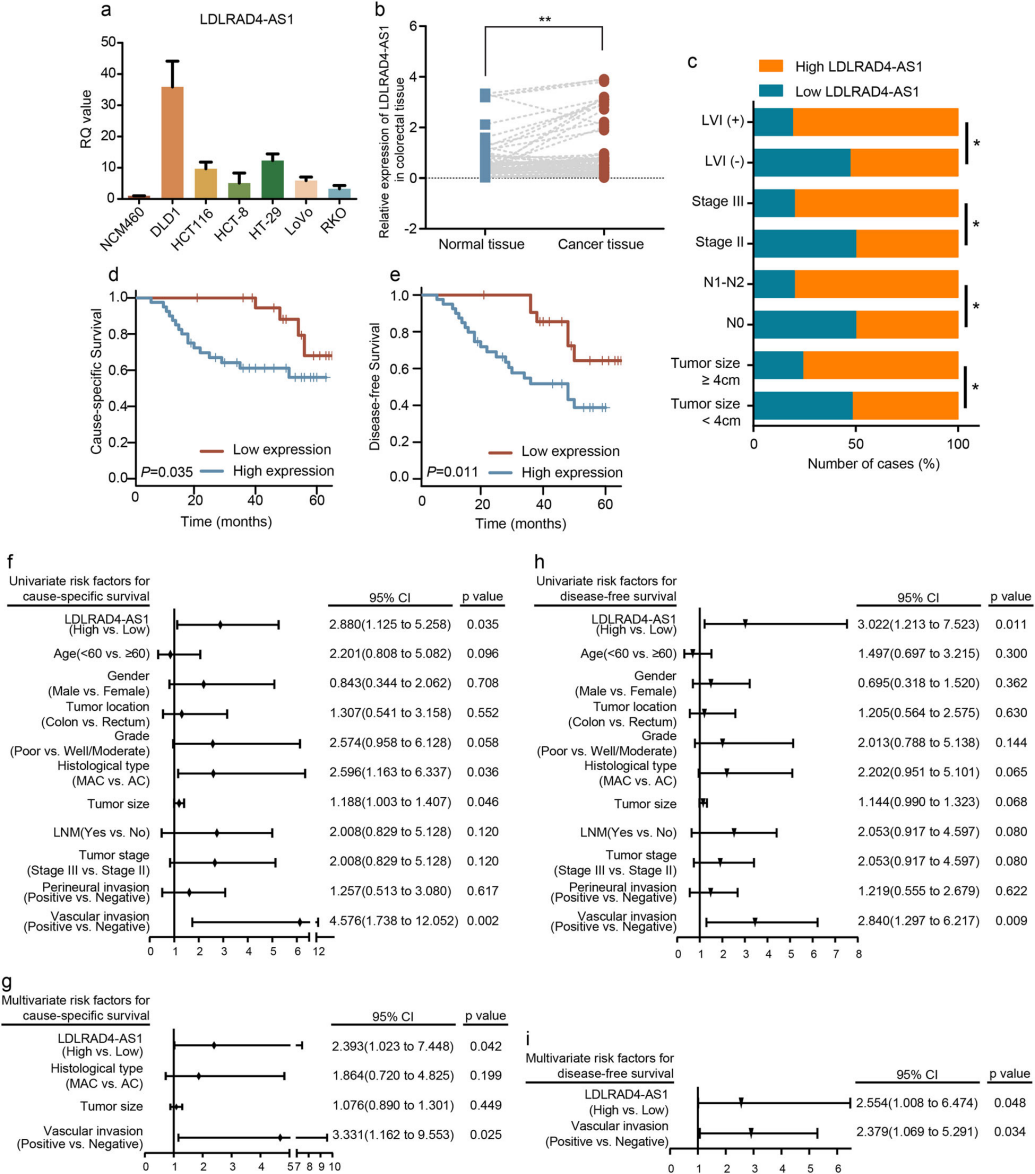
Higher expression of lncRNA LDLRAD4-AS1 was associated with poor prognosis of CRC patients. **a** Expression levels of lncRNA LDLRAD4-AS1 in CRC and colon mucosa epithelial (NCM460) cell lines. **b** Expression levels of lncRNA LDLRAD4-AS1 in 62 paired CRC and adjacent noncancerous tissues. **c** Graphical illustration of statistical lncRNA LDLRAD4-AS1 distribution in CRC patients. **d** Kaplan–Meier analysis of cause-specific survival (CSS) in all patients with CRC according to lncRNA LDLRAD4-AS1 expression (log-rank test). **e** Kaplan–Meier analysis of disease-free survival (DFS) in all patients with CRC according to lncRNA LDLRAD4-AS1 expression (log-rank test). **f** Univariate survival analyses for CSS of patients with lncRNA LDLRAD4-AS1 low and high expression. **g** Multivariate survival analyses for CSS of patients with lncRNA LDLRAD4-AS1 low and high expression. **h** Univariate survival analyses for DFS of patients with lncRNA LDLRAD4-AS1 low and high expression. **i** Multivariate survival analyses for DFS of patients with lncRNA LDLRAD4-AS1 low and high expression. For **a**–**c**, data are presented as means ± SD in three independent experiments. *p < 0.05, **p < 0.01, ***p < 0.001.

To investigate the role of lncRNA LDLRAD4-AS1 in CRC, we first examined the lncRNA LDLRAD4-AS1 expression levels in CRC cell lines, revealing that lncRNA LDLRAD4-AS1 expression was upregulated in all CRC cell lines compared with a normal cell line (NCM460) (*p* < 0.001, Fig. 1a). Then, we further assessed lncRNA LDLRAD4-AS1 expression levels in 62 paired primary CRC tissue samples, and matched adjacent nontumor tissue samples by qRT-PCR. Detailed information on the association between lncRNA LDLRAD4-AS1 and the clinicopathological characteristics of CRC is shown in Table 1. The results indicated that the expression of lncRNA LDLRAD4-AS1 was significantly increased in the CRC tissue samples (*p* = 0.002, Fig. 1b). Moreover, as shown in Table 1 and Fig. 1c, we found that higher expression of lncRNA LDLRAD4-AS1 was significantly correlated with large tumor size (*p* = 0.045), involvement in lymph node metastasis (*p* = 0.014), advanced TNM stage (*p* = 0.014), and vascular invasion (*p* = 0.023) in CRC patients.

**Table 1:** Association between LDLRAD4-AS1 and the clinicopathological characteristics of colorectal cancer.

Variables, *N* (%)	LDLRAD4-AS1 expression		*p*-value
	Low (*N* = 22)	High (*N* = 40)	
*Gender*			0.877
Male	12 (54.5)	21 (52.5)	
Female	10 (45.5)	19 (47.5)	
*Age*			0.195
<60	12 (54.5)	15 (37.5)	
≥60	10 (45.5)	25 (62.5)	
*Tumor location*			0.176
Colon	11 (50.0)	27 (67.5)	
Rectum	11 (50.0)	13 (32.5)	
*Grade*			0.530
Well/moderate	19 (86.4)	32 (80.0)	
Poor	3 (13.6)	8 (20.0)	
*Histological type*			0.417
Adenocarcinoma	17 (77.3)	27 (67.5)	
Mucinous	5 (22.7)	13 (32.5)	
Tumor size (cm)	3.7	5.1	0.045
*Lymph node Metastasis*			0.014
No	16 (72.7)	16 (40.0)	
Yes	6 (27.3)	24 (60.0)	
*Tumor stage*			0.014
Stage II	16 (72.7)	16 (40.0)	
Stage III	6 (27.3)	24 (60.0)	
*Perineural invasion*			0.082
Negative	17 (77.3)	22 (55.0)	
Positive	5 (22.7)	18 (45.0)	
*Vascular invasion*			0.023
Negative	17 (77.3)	19 (47.5)	
Positive	5 (22.7)	21 (52.5)	

Then, to determine the relationship between the expression of lncRNA LDLRAD4-AS1 and CRC patient prognosis, Kaplan–Meier analysis was performed to show that lncRNA LDLRAD4-AS1 expression was markedly associated with a shorter time after surgical resection. The 5-year cause-specific survival (CSS) and disease-free survival (DFS) rates for patients in the high lncRNA LDLRAD4-AS1 expression group were 56.0% and 38.8%, respectively, compared with 68.0% and 64.3% in CRC patients in the low lncRNA LDLRAD4-AS1 expression group (*p* = 0.035, Fig. 1d; *p* = 0.011, Fig. 1e). Moreover, in the univariate (Fig. 1f, h) and multivariate (Fig. 1g, i) Cox proportional hazards models, after adjusting for other effects, lncRNA LDLRAD4-AS1 expression (*p* = 0.042 for CSS, *p* = 0.048 for DFS) and vascular invasion (*p* = 0.025 for CSS, *p* = 0.034 for DFS) were both independently associated with shortened CSS and DFS. These results revealed that higher expression of lncRNA LDLRAD4-AS1 was associated with poor prognosis in CRC patients.

### LncRNA LDLRAD4-AS1 promotes CRC metastasis in vitro and in vivo

**Fig. 2:**
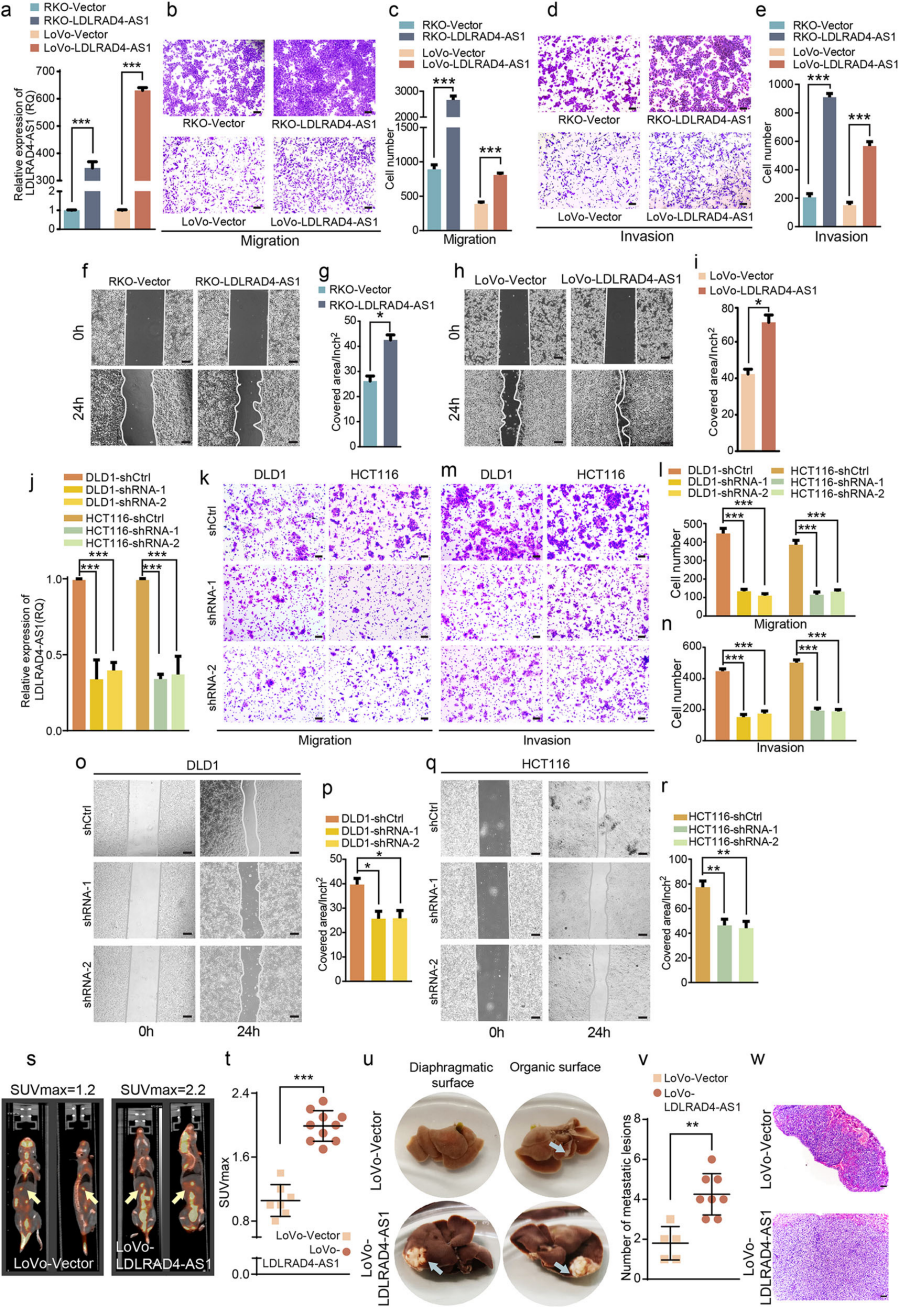
LncRNA LDLRAD4-AS1 promotes CRC cell migration and invasion in vitro as well as promotes CRC metastasis in vivo. **a** LDLRAD4-AS1-overexpressing RKO and LoVo cell lines were established by the transfection of pCDH- LDLRAD4-AS1. LncRNA LDLRAD4-AS1 levels in cells were detected by qRT-PCR. **b**, **c** Migration assays were used to determine the effects of LDLRAD4-AS1 overexpression on the migration ability of CRC cells. **d**, **e** Invasion assays were used to determine the effects of LDLRAD4-AS1 overexpression on the invasion ability of CRC cells. **f**–**i** The migration potencies of CRC cells with the indicated treatments were detected by using wound-healing assay. **j** infecting DLD1 and HCT116 cells with a lentivirus vector harboring shRNA-LDLRAD4-AS1 was to knock down the endogenous expression of lncRNA LDLRAD4-AS1 in cells. lncRNA LDLRAD4-AS1 levels in cells were detected by qRT-PCR. **k**, **l** Migration assays were used to determine the effects of LDLRAD4-AS1-depleted on the migration ability of CRC cells. **m**, **n** Invasion assays were used to determine the effects of LDLRAD4-AS1-depleted on the invasion ability of CRC cells. **o**–**r** The migration potencies of CRC cells with the indicated treatments were detected by using wound-healing assay. **s** Representative photographs of ^18^F-FDG PET/CT scans of LoVo-LDLRAD4-AS1 and control LoVo-Vector-injected mice. **t** The SUVmax was higher in the LoVo-LDLRAD4-AS1 group than in the control group. **u** The gross images of liver metastases observed in the nude mice injected with LoVo cells. Arrows represent metastatic tumors. **v** The number of metastatic lesions was larger in the LoVo-LDLRAD4-AS1 group than in the control group. **w** H&E staining for the liver metastases. For **a**–**w**, data were expressed as means ± SD in three independent experiments. *p < 0.05, **p < 0.01, ***p < 0.001.

Since the high expression of lncRNA LDLRAD4-AS1 hints at a malignant biological behavior of CRC and CRC metastasis is the leading cause of patient death, we investigated the effect of lncRNA LDLRAD4-AS1 on CRC cell metastasis. Using the Transwell assay, we found that the overexpression of lncRNA LDLRAD4-AS1 (Fig. 2a) significantly promoted the migration and invasion of both RKO and LoVo cells in vitro (Fig. 2b–e). Accordingly, the results of the scratch wound-healing assay showed that lncRNA LDLRAD4-AS1 enhanced the migration capacity of RKO and LoVo cells, resulting in significant increases in the covered area in the assays for both cell lines (Fig. 2f–i). Then, we knocked down lncRNA LDLRAD4-AS1 in both DLD1 and HCT116 cells (Fig. 2j). As expected, knockdown of lncRNA LDLRAD4-AS1 led to a significant decrease in the migration and invasion of both cell lines (Fig. 2k–n), in accordance with the results of scratch wound-healing assays performed in DLD1 and HCT116 cells (Fig. 2o–r). To further assess the role of lncRNA LDLRAD4-AS1 in promoting CRC cell metastasis in vivo, we injected LoVo cells with lncRNA LDLRAD4-AS1 overexpression (LoVo-LDLRAD4-AS1) and control cells (LoVo-Vector) into the spleens of nude mice. ^18^F-FDG PET/CT scanning results showed that both LoVo-Vector and LoVo-LDLRAD4-AS1 cells metastasized from the spleens to the livers, while the SUVmax values for the livers of LoVo-LDLRAD4-AS1 cell-injected mice were significantly higher than those of the control mice (Fig. 2s, t). After the mice were euthanized, the livers were resected and observed, and the metastatic lesions were counted, sectioned, and stained with H&E. The results showed that forced expression of LDLRAD4-AS1 significantly increased the number of metastatic lesions on the livers (Fig. 2u–w). The above data demonstrated that LDLRAD4-AS1 could promote the metastasis of CRC cells in vitro and in vivo.

### LncRNA LDLRAD4-AS1 is negatively correlated with LDLRAD4

**Fig. 3:**
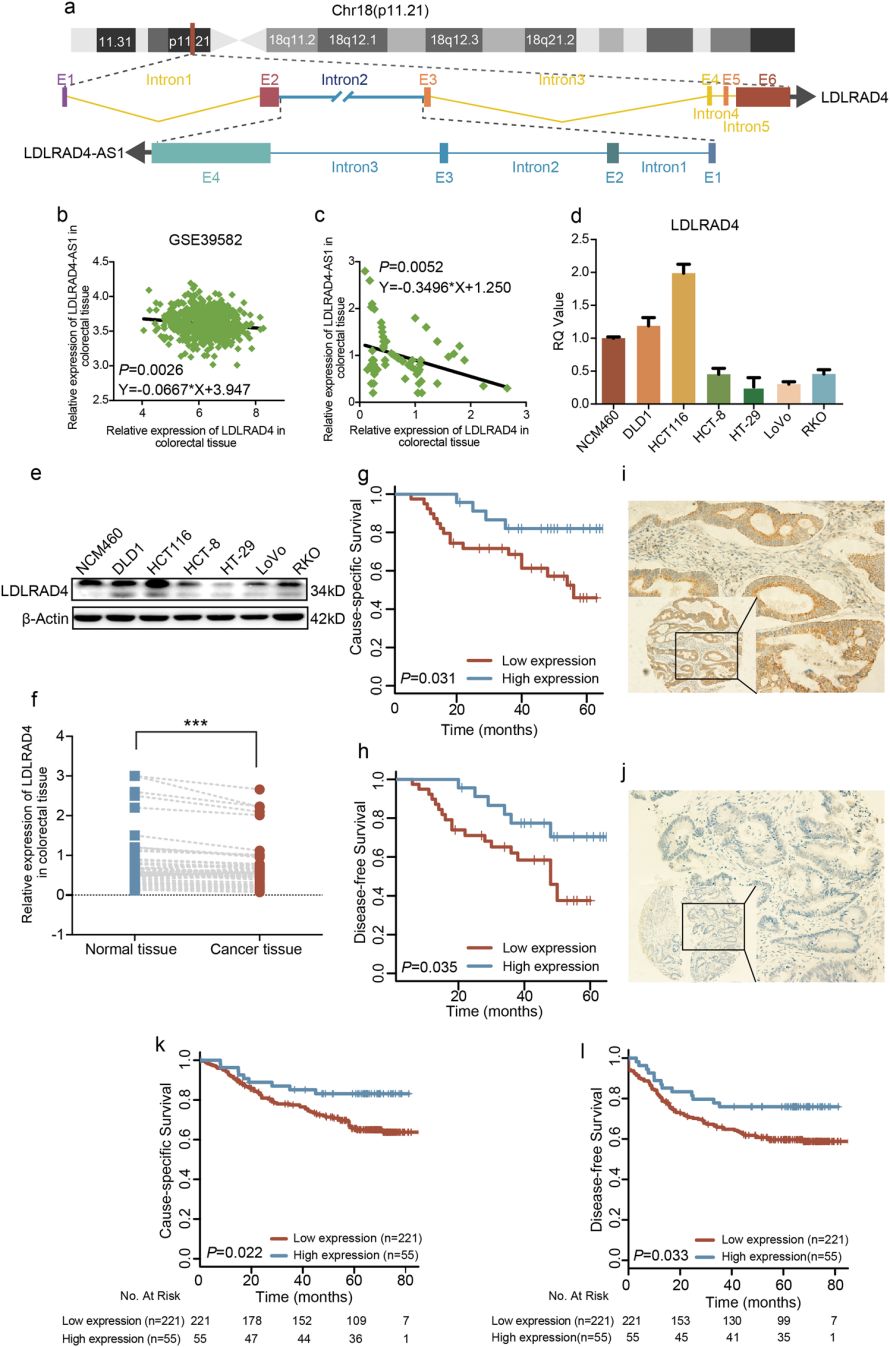
LncRNA LDLRAD4-AS1 negatively correlated with LDLRAD4. **a** lncRNA LDLRAD4-AS1 overlapped with the second intron of LDLRAD4. **b** lncRNA LDLRAD4-AS1 expression was distinctly negatively correlated with LDLRAD4 based on expression data from GSE39582 (Y = −0.0667*X + 3.947, *p* = 0.0026). **c** lncRNA LDLRAD4-AS1 expression was distinctly negatively correlated with LDLRAD4 based on PCR data in the 62 CRC tissues from FUSCC data set (Y = −0.3496*X + 1.250, *p* = 0.0052). **d**, **e** Expression levels of LDLRAD4 in CRC and colon mucosa epithelial (NCM460) cell lines using qRT-PCR analysis (**d**) and western blotting (**e**). **f** Expression levels of LDLRAD4 in 62 paired CRC and adjacent noncancerous tissues. **g** Kaplan–Meier analysis of cause-specific survival (CSS) in all patients with CRC, according to LDLRAD4 expression using qRT-PCR analysis (log-rank test). **h** Kaplan–Meier analysis of disease-free survival (DFS) in all patients with CRC, according to LDLRAD4 expression using qRT-PCR analysis (log-rank test). **i**, **j** Representative images show high LDLRAD4 expression in CRC tissues (**i**) compared with low LDLRAD4 expression in CRC tissues (**j**). **k** Kaplan–Meier analysis of cause-specific survival (CSS) in all patients with CRC, according to LDLRAD4 expression using IHC analysis (log-rank test). **l** Kaplan–Meier analysis of disease-free survival (DFS) in all patients with CRC according to LDLRAD4 expression (log-rank test) using IHC analysis. *p < 0.05, **p < 0.01, ***p < 0.001.

According to the bioinformatics and gene sequence analysis, we found that lncRNA LDLRAD4-AS1 overlapped with the second intron of LDLRAD4 (Fig. 3a), which may lay the structural foundation of the regulatory relationship between the two molecules. To identify any direct association between lncRNA LDLRAD4-AS1 and LDLRAD4 mRNA levels, we investigated lncRNA LDLRAD4-AS1 and LDLRAD4 mRNA expression levels in CRC tissues from the GSE39582 and FUSCC data sets. The analysis revealed a significant negative correlation between lncRNA LDLRAD4-AS1 and LDLRAD4 in both the GSE39582 (Fig. 3b) and FUSCC data sets (Fig. 3c).

Then, to investigate the role of LDLRAD4 in CRC, we first examined LDLRAD4 expression levels in CRC cell lines. Of note, we observed higher LDLRAD4 expression in only two cell lines (DLD1 and HCT116) and lower LDLRAD4 expression in the remaining cell lines, particularly in those with greater invasive ability (RKO and LoVo) (Fig. 3d, e). Moreover, we assessed LDLRAD4 expression levels in 62 paired primary CRC tissue and matched adjacent nontumor tissue samples by qRT-PCR, which demonstrated that the expression of LDLRAD4 was significantly decreased in the CRC tissue samples (*p* < 0.001, Fig. 3f). In addition, Kaplan–Meier analysis showed that LDLRAD4 expression was markedly associated with better survival outcomes. The 5-year CSS and DFS rates for patients in the high LDLRAD4 expression group were 82.0% and 70.4%, respectively, compared with 46.0% and 38.0% in CRC patients in the low LDLRAD4 expression group (*p* = 0.031, Fig. 3g; *p* = 0.035, Fig. 3h). To further investigate the value of LDLRAD4 in CRC cases, we investigated LDLRAD4 protein expression in CRC tissue specimens in the TMA using IHC staining. High and low LDLRAD4 protein expression in CRC tissue specimens is shown in Fig. 3i, j. A description of the study population between CRC patients with low and high LDLRAD4 expression is shown in Table 2. Kaplan–Meier analyses showed that high LDLRAD4 protein expression was associated with better survival outcomes. The 5-year CSS and DFS rates for patients in the high LDLRAD4 protein expression group were 83.1% and 75.9%, respectively, compared with 65.6% and 59.7% in CRC patients in the low LDLRAD4 protein expression group (*p* = 0.022, Fig. 3k; *p* = 0.033, Fig. 3l). Cox regression analysis was performed to test the associations between LDLRAD4 expression and oncologic outcomes. After univariate analysis (Supplementary Table 2), variables with *p* < 0.05 were further evaluated using Cox model multivariate analysis, which indicated that low LDLRAD4 expression, advanced TNM stage, and advanced T stage and M stage were statistically significantly associated with the poor prognosis of CRC patients in terms of both CSS and DFS (Supplementary Table 3). These results demonstrated that lncRNA LDLRAD4-AS1 negatively correlated with LDLRAD4, whose higher expression levels were associated with a better prognosis in CRC patients.

**Table 2:** Description of the study population between colorectal cancer patients with LDLRAD4 low and high expression.

Variables, *N* (%)	LDLRAD4 expression		*p*-value
	Low (*N* = 221)	High (*N* = 55)	
*Gender*			0.555
Male	131 (59.3)	35 (63.6)	
Female	90 (40.7)	20 (36.4)	
*Age*			0.027
<60	129 (58.4)	41 (74.5)	
≥60	92 (41.6)	14 (25.5)	
*TNM stage*			0.139
I	18 (8.1)	3 (5.4)	
II	66 (29.9)	15 (27.3)	
III	99 (44.8)	33 (60.0)	
IV	38 (17.2)	4 (7.3)	
*T stage*			0.461
T2	37 (16.7)	6 (10.9)	
T3	41 (18.6)	13 (23.6)	
T4	143 (64.7)	36 (65.5)	
*N stage*			0.881
N0	98 (44.3)	22 (40.0)	
N1	65 (29.5)	18 (32.7)	
N2	58 (26.2)	15 (27.3)	
*M stage*			0.067
M0	183 (82.8)	51 (92.7)	
M1	38 (17.2)	4 (7.3)	
*Tumor location*			0.489
Colon	101 (45.7)	28 (50.9)	
Rectum	120 (54.3)	27 (49.1)	
*Grade*			0.728
Well/moderate	177 (80.1)	43 (78.2)	
Poor	44 (19.9)	12 (21.8)	
*Histological type*			0.511
Adenocarcinoma	208 (94.1)	53 (96.4)	
Mucinous	13 (5.9)	2 (3.6)	
*LNH*			0.171
<12	69 (31.2)	12 (21.8)	
≥12	152 (68.8)	43 (78.2)	
*Perineural invasion*			0.934
Negative	185 (84.1)	46 (83.6)	
Positive	35 (15.9)	9 (16.4)	
*Vascular invasion*			0.954
Negative	148 (67.0)	36 (65.5)	
Positive	70 (31.7)	18 (32.7)	
Unknown	3 (1.3)	1 (1.8)	
*Pre-treatment CEA*			0.623
Negative	133 (60.2)	37 (67.3)	
Positive	79 (35.7)	16 (29.1)	
Unknown	9 (4.1)	2 (3.6)	
*Adjuvant chemotherapy*			0.026
No	41 (18.5)	8 (14.6)	
Yes	144 (65.2)	45 (81.8)	
Unknown	36 (16.3)	2 (3.6)	
*MSI status/MMR status*			0.004
MSS/MMR-proficient	150 (67.9)	26 (47.3)	
MSI/MMR-deficient	71 (32.1)	29 (52.7)	

*LNH* number of lymph nodes harvested, *CEA* carcinoembryonic antigen, *MSI* microsatellite instability, *MSS* microsatellite stability, *MMR* mismatch repair.

### LDLRAD4 acts as a metastasis suppressor in CRC

**Fig. 4:**
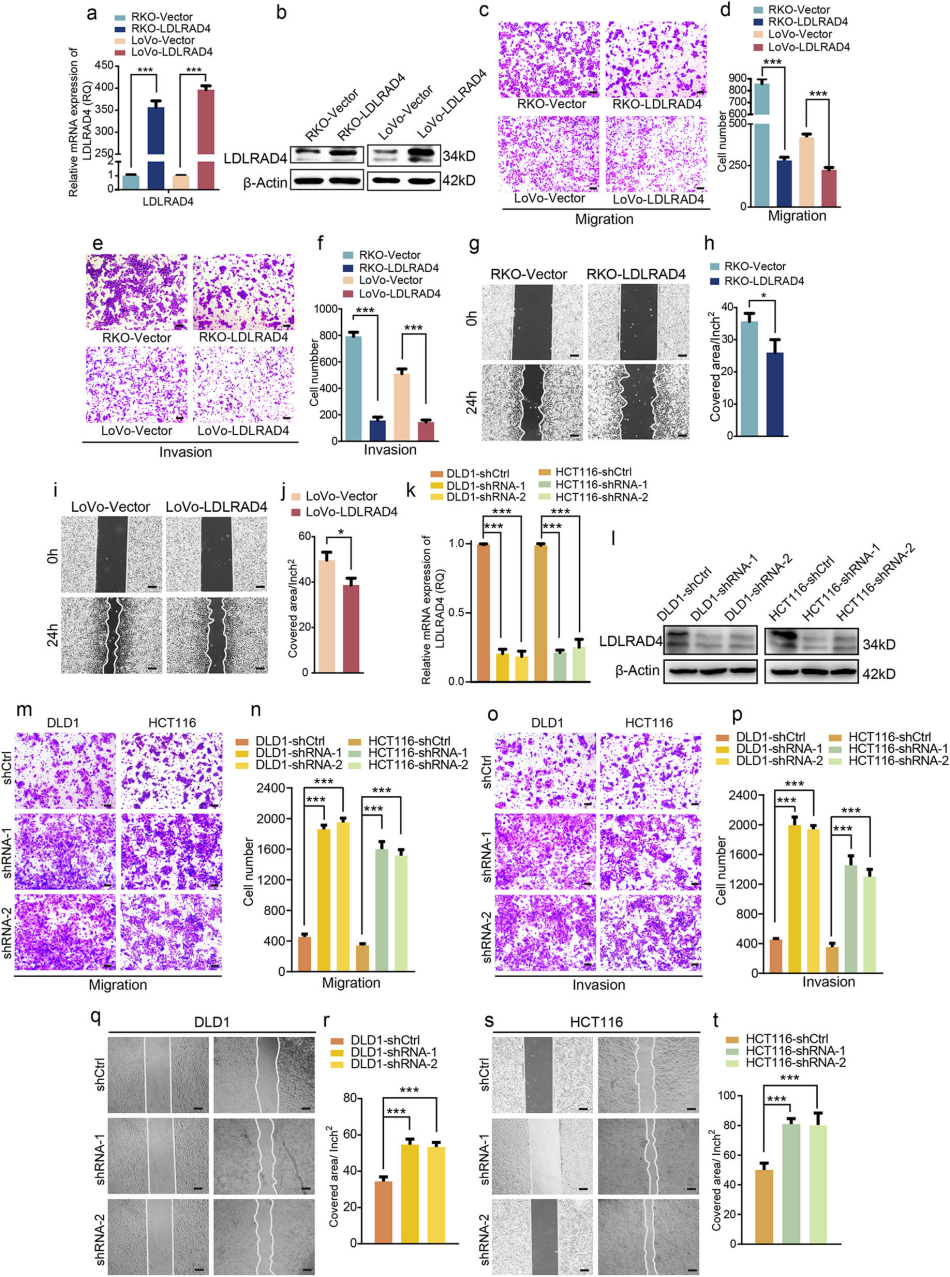
LDLRAD4 acts as a metastasis suppressor in CRC. **a**, **b** LDLRAD4-overexpressing RKO and LoVo cell lines were established by the transfection of pCDH-LDLRAD4. LDLRAD4 levels in cells were detected by qRT-PCR (**a**) and western blotting (**b**). **c**, **d** Migration assays were used to determine the effects of LDLRAD4 overexpression on the migration ability of CRC cells. **e**, **f** Invasion assays were used to determine the effects of LDLRAD4 overexpression on the invasion ability of CRC cells. **g**–**j** The migration potencies of CRC cells with the indicated treatments were detected by using wound-healing assay. **k**–**l** infecting DLD1 and HCT116 cells with a lentivirus vector harboring shRNA-LDLRAD4 was to knock down the endogenous expression of LDLRAD4 in cells. LDLRAD4 levels in cells were detected by qRT-PCR (**k**) and western blotting (**l**). **m**, **n** Migration assays were used to determine the effects of LDLRAD4-depleted on the migration ability of CRC cells. **o**, **p** Invasion assays were used to determine the effects of LDLRAD4-depleted on the invasion ability of CRC cells. **q**–**t** The migration potencies of CRC cells with the indicated treatments were detected by using wound-healing assay. For **a**–**t**, data were expressed as means ± SD in three independent experiments. *p < 0.05, **p < 0.01, ***p < 0.001.

To reveal whether the negative correlation between lncRNA LDLRAD4-AS1 and LDLRAD4 determines the metastasis-promoting role of lncRNA LDLRAD4-AS1, we detected the role of LDLRAD4 in CRC cell metastasis in vitro. Overexpression of LDLRAD4 (Fig. 4a, b) significantly impeded the migration and invasion of RKO and LoVo cells in vitro, as revealed by Transwell (Fig. 4c–f) and scratch wound-healing assays (Fig. 4g–j). Further, we knocked down LDLRAD4 to confirm its metastasis-inhibiting role (Fig. 4k, l). As shown in Fig. 4m–p, the silencing of LDLRAD4 significantly promoted the migration and invasion of DLD1 and HCT116 cells in vitro. In addition, the decrease in LDLRAD4 brought about a significant increase in the covered area in the scratch wound-healing assays for both DLD1 and HCT116 cells (Fig. 4q–t). These results concordantly indicate that LDLRAD4 acts as a CRC metastasis suppressor, and it could be speculated that the negative correlation between LDLRAD4-AS1 and LDLRAD4 might be the underlying mechanism of the metastasis-promoting role of lncRNA LDLRAD4-AS1.

### LncRNA LDLRAD4-AS1 disrupts the stability of LDLRAD4 mRNA

**Fig. 5:**
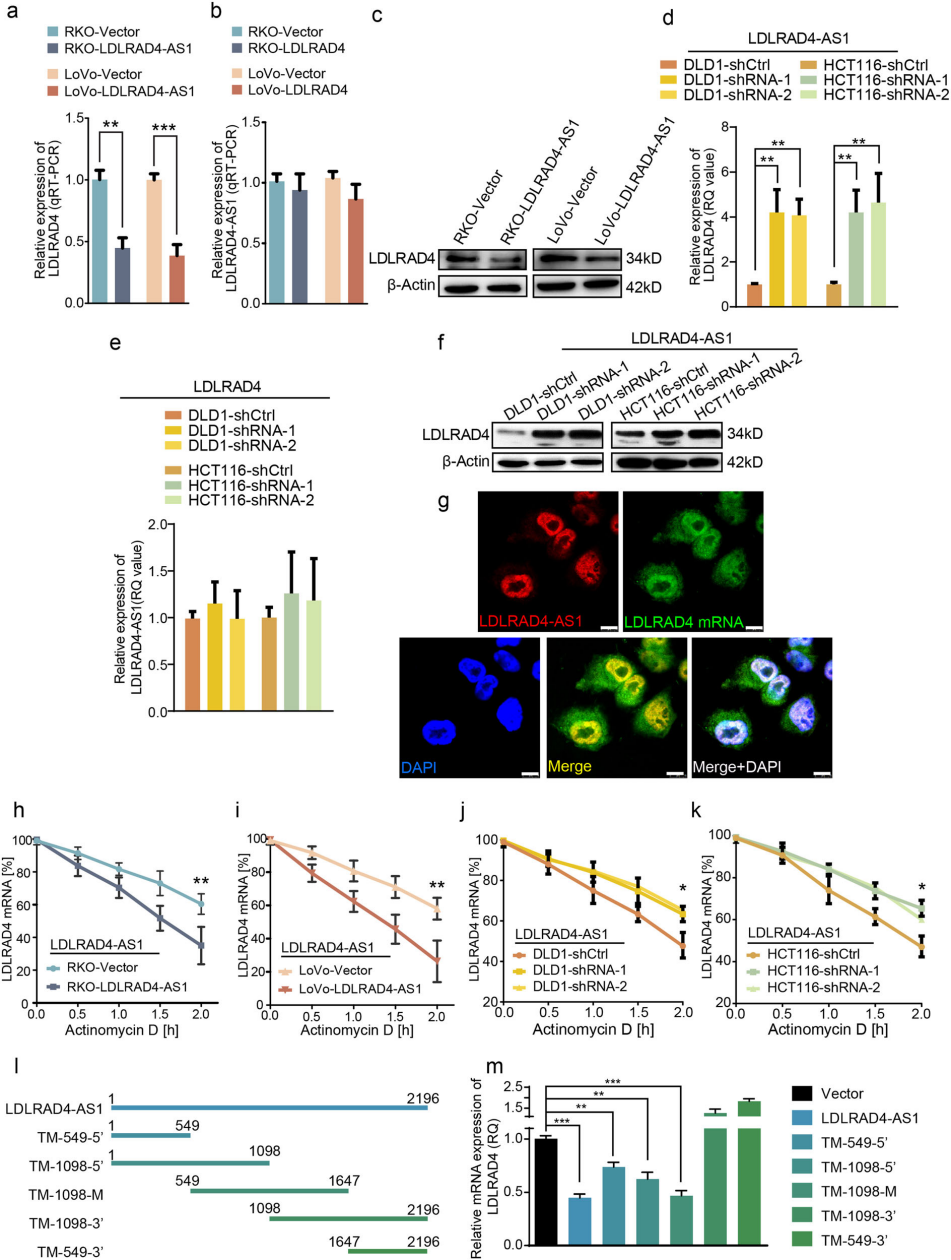
LncRNA LDLRAD4-AS1 disrupts the stability of LDLRAD4 mRNA. **a** Expression levels of LDLRAD4 in LDLRAD4-AS1-overexpression cells using qRT-PCR analysis. **b** Expression levels of lncRNA LDLRAD4-AS1 in LDLRAD4-overexpression cells using qRT-PCR analysis. **c** Expression levels of LDLRAD4 in LDLRAD4-AS1-overexpression cells using western blotting. **d** Expression levels of LDLRAD4 in LDLRAD4-AS1-depleted cells using qRT-PCR analysis. **e** Expression levels of lncRNA LDLRAD4-AS1 in LDLRAD4-depleted cells using qRT-PCR analysis. **f** Expression levels of LDLRAD4 in LDLRAD4-AS1-depleted cells using western blotting. **g** RNA fluorescence in situ hybridization (FISH) showed that lncRNA LDLRAD4-AS1 and LDLRAD4 were both located in the nucleus in close proximity to each other. **h**–**k** LncRNA LDLRAD4-AS1 overexpression disrupted stability of LDLRAD4 mRNA compared with the control group (**h**–**i**), whereas the stability of LDLRAD4 mRNA was obviously increased after lncRNA LDLRAD4-AS1 knockdown compared with the control group (**j**, **k**). **l** All five truncated mutants and lncRNA LDLRAD4-AS1 full length are illustrated. **m** Forced expression of three truncated mutants (TM-549-5′, TM-1098-5′, and TM-1098-M) significantly downregulated the levels of LDLRAD4 mRNA as lncRNA LDLRAD4-AS1 full length did. For **a**–**m**, data were expressed as means ± SD in three independent experiments. *p < 0.05, **p < 0.01, ***p < 0.001.

Given the negative correlation between the expression of lncRNA LDLRAD4-AS1 and LDLRAD4 and the metastasis suppressor role of LDLRAD4, we sought to uncover the mechanism by which lncRNA LDLRAD4-AS1 promotes CRC metastasis by analyzing its role in regulating LDLRAD4. We first investigated the effect of lncRNA LDLRAD4-AS1 on the expression of LDLRAD4. Overexpression of lncRNA LDLRAD4-AS1 significantly decreased the mRNA level and the protein level of LDLRAD4 (Fig. 5a, c), while the elevated expression of LDLRAD4 did not cause obvious changes to the level of lncRNA LDLRAD4-AS1 (Fig. 5b) in either RKO or LoVo cells. In addition, knockdown of lncRNA LDLRAD4-AS1 increased the mRNA and protein levels of LDLRAD4 in DLD1 and HCT116 cells (Fig. 5d, f). The knockdown of LDLRAD4 did not significantly change the level of lncRNA LDLRAD4-AS1 in DLD1 and HCT116 cells (Fig. 5e).

Since lncRNA LDLRAD4-AS1 showed the ability to downregulate LDLRAD4 mRNA, we hypothesized that it could regulate the stability by interacting with LDLRAD4 mRNA. Using fluorophore-labeled probes, we detected the intracellular localization of lncRNA LDLRAD4-AS1 and LDLRAD4 mRNA. As shown in Fig. 5g, lncRNA LDLRAD4-AS1 is predominantly located in the nucleus, and LDLRAD4 mRNA is located in both the nucleus and cytoplasm. After merging the images, lncRNA LDLRAD4-AS1 and LDLRAD4 mRNA showed colocalization in the nucleus (Fig. 5g), which makes them available to interact with each other. Then, we applied an RNA stability assay to detect the effect of lncRNA LDLRAD4-AS1 on the stability of LDLRAD4 mRNA. After blocking RNA transcription by adding actinomycin D, LDLRAD4 mRNA exhibited an obvious decrease in its stability mediated by lncRNA LDLRAD4-AS1 in both RKO and LoVo cells (Fig. 5h, i). In accordance, knockdown of lncRNA LDLRAD4-AS1 extended the half-life of LDLRAD4 mRNA in DLD1 and HCT116 cells (Fig. 5j, k). Furthermore, to explore the sequence of lncRNA LDLRAD4-AS1 that is critical for the disruption of LDLRAD4 mRNA, we established plasmids expressing truncated mutants of lncRNA LDLRAD4-AS1 (Fig. 5l). Then, we detected the levels of LDLRAD4 mRNA in 293T cells transiently expressing these truncated mutants. The results showed that forced expression of three truncated mutants (TM-549-5′, TM-1098-5′, and TM-1098-M) significantly downregulated the levels of LDLRAD4 mRNA in the same manner as full-length lncRNA LDLRAD4-AS1 (Fig. 5m). As shown in Fig. 5m, we found that the critical sequence regulating LDLRAD4 mRNA stability in LDLRAD4-AS1 is located in the 1–1098 bp region. The above data demonstrate that lncRNA LDLRAD4-AS1 negatively correlates with LDLRAD4 by disrupting the stability of LDLRAD4 mRNA.

### LncRNA LDLRAD4-AS1 promotes the EMT process in vitro and in vivo

**Fig. 6:**
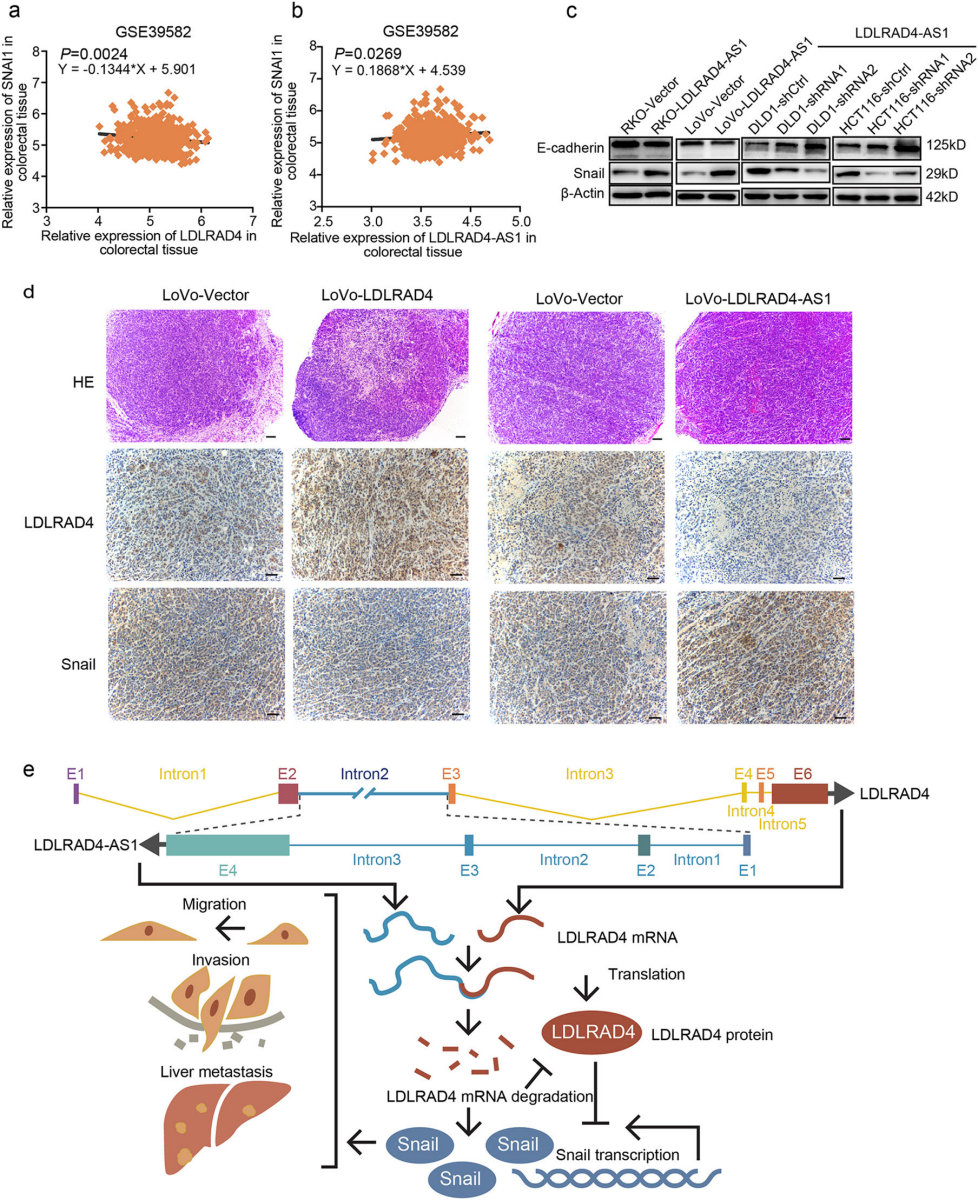
LncRNA LDLRAD4-AS1 promotes the EMT process in vitro and in vivo. **a** LDLRAD4 expression was distinctly negatively correlated with SNAI1 based on expression data from GSE39582 (Y = −0.1344*X + 5.901, *p* = 0.0024). **b** LncRNA LDLRAD4-AS1 expression was distinctly positively correlated with SNAI1 based on expression data from GSE39582 (Y = 0.1868*X + 4.539, *p* = 0.0269). **c** Snail increased with lncRNA LDLRAD4-AS1 overexpression and decreased with lncRNA LDLRAD4-AS1 silencing using western blotting, and E-cadherin changed accordingly. Also, snail decreased with LDLRAD4 overexpression and increased with LDLRAD4 silencing using western blotting, and E-cadherin changed accordingly. **d** The images of H&E staining, IHC for LDLRAD4 and snail in xenografts were shown. **e** A proposed model for illustrating the function and mechanism of lncRNA LDLRAD4-AS1 in CRC EMT and metastasis.

To further explore the mechanism by which lncRNA LDLRAD4-AS1 promotes CRC cell metastasis, we assessed the association between the expression of LDLRAD4 and that of the mastery regulator of EMT, snail, by analyzing the data from GSE39582. The analysis revealed a significant negative correlation between them (Fig. 6a). As expected, lncRNA LDLRAD4-AS1 expression showed a significant positive correlation with snail expression (Fig. 6b). To confirm the correlation between lncRNA LDLRAD4-AS1 and EMT, we performed western blotting to detect snail and E-cadherin. The results showed that snail increased with lncRNA LDLRAD4-AS1 overexpression and decreased with lncRNA LDLRAD4-AS1 silencing, and E-cadherin changed accordingly (Fig. 6c). Furthermore, to determine whether the association between lncRNA LDLRAD4-AS1, LDLRAD4, and snail was be maintained in vivo, we stained for LDLRAD4 and snail in the xenografts by IHC. The results showed that overexpression of LDLRAD4 decreased the protein level of snail, and the elevation of lncRNA LDLRAD4-AS1 decreased the protein level of LDLRAD4 and increased the protein level of snail in vivo (Fig. 6d). These results illustrated that lncRNA LDLRAD4-AS1 could promote the EMT process in vitro and in vivo. Collectively, these results suggest that lncRNA LDLRAD4-AS1 promotes EMT and metastasis by decreasing the expression of LDLRAD4 (Fig. 6e).

## Discussion

The main finding of this study is that the lncRNA LDLRAD4-AS1 is expressed in the nucleolus of CRC cells and is commonly upregulated in most CRC specimens and CRC cell lines. A high level of lncRNA LDLRAD4-AS1 expression is associated with an advanced stage and a poor prognosis in CRC patients. Moreover, high expression of lncRNA LDLRAD4-AS1 contributed to CRC cell invasion and metastasis. Notably, the upregulation of lncRNA LDLRAD4-AS1 expression disrupted the stability of LDLRAD4 mRNA, resulting in EMT and promoting metastasis. Therefore, our results reveal a novel mechanism in which the LDLRAD4-AS1/LDLRAD4/EMT regulatory axis is involved in CRC progression and metastasis, suggesting that lncRNA LDLRAD4-AS1 could be a potential biomarker and treatment target in CRC.

Metastatic spread occurs in ~60% of CRC patients, resulting in poor outcomes, with a median overall survival of ~24–27 months and a 10–15% 5-year survival rate^21^. In recent years, an increasing number of studies have been devoted to the study of prognostic molecular markers of CRC^22^. Accumulating evidence has revealed that lncRNAs play a critical role in prognosis and could be therapeutic targets in CRC^23^. Overexpression of lncRNA FEZF1-AS1 was associated with advanced T stage, lymph node metastasis, distant metastasis and poor overall survival in CRC^24,25^. In contrast, lower expression of lncRNA HOXA11-AS was strongly linked with tumor size, advanced TNM stage, and lymph node metastasis in CRC patients^26^. In our study, overexpression of lncRNA LDLRAD4-AS1 was related to advanced stage, lymph node metastasis, tumor size, vascular invasion, and poor prognosis in CRC. Furthermore, we found that lncRNA LDLRAD4-AS1 is an independent prognostic factor in CRC patients that could act as a potential biomarker and treatment target in CRC.

In addition, the liver is the most common site of CRC metastasis due to the portal venous system, and liver metastasis occurs in ~50% of patients with primary CRC^27^. In our study, we found that patients with overexpression of lncRNA LDLRAD4-AS1 were more prone to liver metastasis. Interestingly, lncRNA LDLRAD4-AS1 was significantly correlated with vascular invasion in terms of clinicopathological features, and lncRNA LDLRAD4-AS1 and vascular invasion were both independent risk factors for prognosis in CRC after both univariate and multivariate analyses.

The potential molecular mechanisms of CRC metastasis have been extensively studied in the last few decades. Increasing evidence shows that lncRNAs regulate the molecular processes of tumors in many aspects, such as transcription, translation, transcriptional interference, epigenetic modification, and cell cycle control^16,28^. Mechanistically, lncRNA HOXD-AS1 serves as a competing endogenous RNA (ceRNA) for miR-217 and increases the expression of AEG-1 and EZH2, which promotes CRC cell proliferation and invasion in vitro, as well as EMT and metastasis in vivo^29,30^. LncRNA SNHG1 regulates β-catenin, transcription factor-4 (TCF-4), cyclin D1, and MMP-9 expression through activation of the WNT/β-catenin pathway, whose downregulation may reduce proliferation, migration and invasion of CRC cells^31,32^. LncRNA FEZF1-AS1 can bind with pyruvate kinase 2 (PKM2) and maintain its stability to increase cytoplasmic and nuclear PKM2; increased cytoplasmic PKM2 enhances pyruvate kinase activity and increases lactate production (aerobic glycolysis), whereas nuclear PKM2 upregulation further activates signal transducer and activator of transcription 3 (STAT3)^33,34^. Zhang et al.^18^ found that lncRNA FOXC2-AS1 exerts its functions by forming an RNA–RNA duplex with FOXC2, thereby increasing FOXC2 at both the mRNA and protein levels and further facilitating the expression of the classical multidrug-resistance gene ABCB1. In this study, we detected that lncRNA LDLRAD4-AS1 and LDLRAD4 mRNA colocalized in the nucleus, and the upregulation of lncRNA LDLRAD4-AS1 expression disrupted the stability of LDLRAD4 mRNA and decreased its protein levels, resulting in EMT and promoting metastasis. Furthermore, we also found that the critical sequence regulating LDLRAD4 mRNA stability in LDLRAD4-AS1 is located in the 1–1098 bp region.

EMT is a dynamic process that allows epithelial cells to acquire mesenchymal features and is mediated by a set of EMT-activating transcription factors (EMT-TFs), which contains three protein families, including Snail^35^. Previous research revealed that the distinct cellular localization of a lncRNA determines its molecular function^36^. Nuclear lncRNAs have functions at the transcriptional level, such as histone modification, alternative splicing, or direct transcriptional regulation^37^. In our study, the upregulation of lncRNA LDLRAD4-AS1 expression disrupted the stability of LDLRAD4 mRNA and decreased its protein levels, resulting in increased snail and decreased E-cadherin, which promotes EMT in CRC. These findings demonstrate that dysregulated expression of lncRNA LDLRAD4-AS1 could serve as a biomarker or therapeutic target in CRC.

In conclusion, we established a previously unknown function for the nuclear lncRNA LDLRAD4-AS1 in CRC. The effects of lncRNA LDLRAD4-AS1 on cell migration and invasion suggest that lncRNA LDLRAD4-AS1 promotes the tumorigenesis and progression of CRC. Moreover, we also provide evidence that LDLRAD4 may represent a downstream effector of lncRNA LDLRAD4-AS1. LncRNA LDLRAD4-AS1 expression disrupted the stability of LDLRAD4 mRNA, resulting in EMT, thereby promoting metastasis. These results may contribute to the development of lncRNA LDLRAD4-AS1-based therapeutic strategies, providing a novel therapeutic approach for CRC treatment.

## Materials and methods

### GEO database, CRC patient cohort, and patient information

We obtained raw microarray colon cancer data sets from the GEO database (http://www.ncbi.nlm.nih.gov/geo/), which were normalized using Robust Multichip Average^38^. The data sets were produced by the Affymetrix HG-U133 plus 2.0 array. All probes were mapped based on their own Entrez GeneID. When multiple probes were mapped to the same Entrez GeneID, the mean value was used to reflect its average expression level. We obtained the clinical parameters and lncRNA LDLRAD4-AS1, LDLRAD4, and SNAI1 expression levels from the GSE39582 data set since it was the largest data set consisting of 497 stage I–III colon cancer patients.

### Fudan University Shanghai Cancer Center (FUSCC) data set

A total of 62 primary CRC patients who received radical colorectal surgery at FUSCC between 2008 and 2009 were included in this study. The Ethical Committee of FUSCC reviewed and approved this study design. All eligible patients signed written informed consent. The enrollment criteria included (a) pathologically confirmed primary CRC and no history of other cancers; (b) planned curative surgical resection; and (c) complete clinical and follow-up data. Depending on the follow-up system of FUSCC, the clinical statistics center of the hospital provided survival data. Once separated, the resected specimens were immediately put into liquid nitrogen and transferred to −80 °C for preservation. The total RNA was isolated by using TRIzol reagent (10296010, Thermo Fisher Scientific (Waltham, MA, USA)), according to the manufacturer’s instructions. SYBR Green Supermix (Takara) was used to perform real-time polymerase chain reaction (PCR) on a QuantStudio™ 7 Flex Real-Time PCR System platform (Thermo Fisher Scientific (Waltham, MA, USA)) following standard protocols.

### Tissue microarray (TMA) construction and immunohistochemistry (IHC) staining

Construction of the TMA used in this study has been previously described in detail^39^. LDLRAD4 anti-human rabbit polyclonal antibody was used at a dilution of 1:200 (bs-9652R, BIOSS); PBS was used as a negative control. Every sample was scored independently by two pathologists utilizing a semiquantitative scoring system that contained the staining intensity scored as 0 (negative), 1 (weak), 2 (medium), or 3 (strong), and the percentage of positive staining areas scored as 0 (<5%), 1 (5–25%), 2 (26–50%), 3 (51–75%), and 4 (>75%). Then, the staining intensity score and the percentage of positive staining area score were multiplied to generate the immunoreactivity score (IRS) for each sample. High expression of LDLRAD4 was defined as detectable immunoreactions in the cytoplasm and cytomembrane with IRS > 4.

### Antibodies and reagents

The following antibodies and reagents were used: anti-β-actin antibody (sc-47778, Santa Cruz Biotechnology), anti-LDLRAD4 antibody (bs-9652R, BIOSS), anti-Ki67 antibody (ab833, Abcam), anti-Snail antibody (ab53519, Abcam), and anti-E-cadherin antibody (20874-1-AP, Proteintech). HRP-conjugated secondary antibodies goat anti-mouse IgG (H + L) (SA00001–1), and goat anti-rabbit IgG (H + L) (SA00001–2) were purchased from Proteintech Group (Rosemont, IL, USA). Fluorescent secondary antibodies [goat anti-rabbit IgG (H + L) highly cross-adsorbed secondary antibody, Alexa Fluor 488 (A-11034) and goat anti-mouse IgG (H + L) highly cross-adsorbed secondary antibody, Alexa Fluor 594 (A-11032)] and lipofectamine 3000 (L3000015) were purchased from Thermo Fisher Scientific (Waltham, MA, USA). Puromycin (P8230) was purchased from Solarbio Biotechnology (Shanghai, China).

### Cell culture and stable cell line establishment

The human colon cancer cell lines (RKO, LoVo, DLD1, HCT116, HCT-8, and HT-29 (Type Culture Collection Cell Bank, Chinese Academy of Sciences)), 293T (Type Culture Collection Cell Bank, Chinese Academy of Sciences), and NCM460 (the American Type Culture Collection (Manassas, VA)) used for cell experiments were cultured in medium supplemented with 10% FBS (Gibco, Life Technology, Austria) and 1% antibiotics, and maintained at 37 °C in a humidified atmosphere containing 5% CO_2_ in a cell incubator (Thermo Fisher Scientific). Full-length LDLRAD4 and lncRNA LDLRAD4-AS1 cDNAs were cloned into the pCDH-CMV-MCS-EF1-Puro vector to generate pCDH-LDLRAD4 and pCDH-LDLRAD4-AS1 expression vectors, respectively. The shRNA plasmids targeting LDLRAD4/LDLRAD4-AS1 were purchased from Shanghai Genechem. Stably transfected CRC cells were established using puromycin selection after transfection with an expression vector or a control plasmid.

### Quantitative real-time PCR

The total RNA was isolated by using TRIzol reagent (10296010, Thermo Fisher Scientific (Waltham, MA, USA)), according to the manufacturer’s instructions. SYBR Green Supermix (Takara) was used to perform real-time polymerase chain reaction (PCR) on a QuantStudio™ 7 Flex Real-Time PCR System platform (Thermo Fisher Scientific (Waltham, MA, USA)) following standard protocols. The expression of β-actin was set as an endogenous control, and the expression of genes was calibrated to that of the corresponding control cells. Data analyses were conducted by QuantStudio Real-Time PCR Software. RQ values (relative quantified value of mRNA expression) were calculated by the same software and analyzed by two-tailed Student’s *t* test to determine significant differences between two groups. All primers designed for qRT-PCR are listed in Supplementary Table 1.

### Western blotting

RIPA lysis buffer was used for protein extraction. Protein was separated by SDS-PAGE (6–20% gel) and then transferred to the PVDF membranes. Western blotting was performed using primary antibodies against LDLRAD4 (bs-9652R, 1:1000, BIOSS), β-actin (sc-47778, 1:500, Santa Cruz Biotechnology), Snail (ab53519, 1:1000, Abcam), and E-cadherin (20874-1-AP, 1:1000, Proteintech), and a secondary antibody (anti-rabbit IgG, 1:7500, Cell Signaling Technology). Immunoreactive proteins were detected by ECL (Pierce, Thermo Scientific) using a Bioimaging System after incubation with species-specific horseradish peroxidase-conjugated secondary antibodies. β-Actin served as the loading control.

### RNA fluorescence in situ hybridization (FISH)

FISH was performed according to the manufacturer’s protocol (Thermo Fisher). After hybridization of the probes, the cells were washed, and the coverslips were mounted with mounting medium containing DAPI for imaging. Slides were observed, and images were captured with a Leica TCS-SP5 confocal system. The LDLRAD4-AS1 probe was 5′-CY3-TGCTTCCTGGGCTTCACAGAGGGTAACAAATGAC-CY3-3′. The LDLRAD4 probe was 5′-FAM-GAGTTCCATCTGCTGTTCAGGGTCC-FAM-3′.

### Transwell migration and invasion assays

For the cell migration assay, cells were harvested, washed twice with PBS, resuspended in DMEM without FBS and counted by a Vi-CELL XR counter (Beckman Coulter). Transwell (8 μm, 353097, Corning) chambers were inserted into the corresponding wells in a 24-well plate that already contained 500 μL DMEM with 10% FBS. Cells at a density of 2 × 10^4^ cells/well were placed into the upper chamber and incubated for 48 h. For the cell invasion assay, cells at a density of 5 × 10^4^ cells/well were placed in serum-free medium in the upper well of 24-well Transwell inserts coated with Matrigel and incubated for 48 h. For both the cell migration assay and invasion assay, following incubation, the medium of the upper chambers was discarded, and migrated cells on the lower side were fixed and stained with 4% paraformaldehyde at room temperature for 30 min, stained with 0.5% crystal violet at room temperature for 30 min, and washed with PBS three times. Cells were observed, and images were captured with an Olympus microscope system and then counted in three out of eight randomly chosen, equally divided areas, followed by two-tailed Student’s *t* test to determine significant differences between two groups.

### Wound-healing assays

Cells were cultured in growth medium in six-well plates. After 24 h to reach complete confluency, a scratch wound was created with a pipette tip on confluent cells in the center of the plate as the starting point, and images of cell migration into the wound were captured at 0, 24, and 48 h using a light microscope. The results are expressed as follows: covered area (inch^2^).

### mRNA decay analyses

Stable cells were directly harvested (mRNA steady-state level) or treated with 5 mM actinomycin D (mRNA decay) and harvested at the indicated time points. The total RNA was isolated at the indicated time points after actinomycin D application, and each analyzed factor was validated by quantitative RT-PCR for each experiment. All data were analyzed from at least three independent experiments, and statistical significance was validated by Student’s *t* test.

### Establishment of lncRNA LDLRAD4-AS1 truncated mutants and identification of critical sequence

To locate the critical sequence for LDLRAD4 mRNA disruption in lncRNA LDLRAD4-AS1, we established five truncated mutants of lncRNA LDLRAD4-AS1. Based on the full-length lncRNA LDLRAD4-AS1 sequence (2196 bp), we established truncated mutants as follows (5′–3′): mutant TM-549-5′ (TM indicates truncated mutant) is the 1–549 bp region, mutant TM-1098–5′ is the 1–1098 bp region, mutant TM-1098-M is the 549–1647 bp region, mutant TM-1098–3′ is the 1098–2196 bp region, and mutant TM-549-3′ is the 1647–2196 bp region. All five truncated mutants and full-length lncRNA LDLRAD4-AS1 are illustrated in Fig. 5l. The sequences were cloned into the pCDH-CMV-MCS-EF1-Puro empty vector and sequenced. Then, the recombinant plasmids were transiently transfected into 293T cells using HighGene Transfection reagent (ABclonal, Shanghai, China). Forty-eight hours after transfection, RNA was harvested and reverse transcribed into cDNA. qRT-PCR was applied to detect the levels of LDLRAD4 mRNA.

### Xenotransplant murine models and PET/CT study

CRC cells (5 × 10^6^ cells/mouse) were suspended in 100 μL Matrigel, injected subcutaneously into the right flank of nude mice (*n* = 6, male; 5-week-old Balb/C athymic nude mouse), and allowed to grow for 4 weeks. Tumor growth was monitored using calipers every 4 days. Four weeks after tumor cell injection, animals were euthanized when tumors reached ~10% of body weight. Primary tumors and organs were harvested and fixed in 10% formalin and paraffin embedded for pathological analysis. For the metastasis assay, stable LoVo-LDLRAD4-AS1 or vector cells (1 × 10^6^ cells) were implanted into the spleens of nude mice and allowed to grow for 6 weeks. All animal studies were conducted in accordance with the animal care guidelines at FUSCC. For the PET/CT study, the mice were starved for 8 h, then given 6 µCi ^18^F-FDG per gram body weight and subjected to a PET/CT scan 1 h later.

### Statistical analysis

Data from in vitro experiments are presented as the mean ± SD, and the difference was analyzed using one-way ANOVA or Student’s *t* test. Fisher’s exact test or a two-tailed χ^2^ test was used to define the relationship between the expression levels of the LDLRAD4 or lncRNA LDLRAD4-AS1 gene and patient characteristics parameters. Survival analysis was performed using the Kaplan–Meier method, and a log-rank test was used to determine the significance of the differences in survival. All statistical analyses were performed using SPSS software (version 22.0; SPSS, Chicago, IL) and GraphPad Prism 6 (La Jolla, CA, USA). All confidence intervals (CIs) were stated at the 95% confidence level. *P*-values < 0.05 were considered statistically significant.

## Supplementary information

Supplementary figure and table legends

Supplementary Table 1

Supplementary Table 2

Supplementary Table 3

Supplementary Figure 1

## Data Availability

The data sets used and/or analyzed during the current study are available from the corresponding author on reasonable request.
