# Supplementary material for: Antisense lncRNA LDLRAD4-AS1 promotes metastasis by decreasing the expression of LDLRAD4 and predicts a poor prognosis in colorectal cancer
Source: Cell Death Dis. 2020 Feb 28;11(2):155. doi: 10.1038/s41419-020-2338-y (PMC7048743; doi:10.1038/s41419-020-2338-y)
Supplement: Supplementary file 1 — Supplementary figure and table legends [file 41419_2020_2338_MOESM1_ESM.docx]

**Additional files**

**Supplementary Table 1:** Primers used for PCR validation.

**Supplementary Table 2:** Univariate survival analyses of patients with LDLRAD4 low and high expression.

**Supplementary Table 3:** Multivariate survival analyses of patients with LDLRAD4 low and high expression.

**Supplementary Figure 1:** Expression levels of 8 potential antisense lncRNAs in 20 paired CRC tumor tissues and its adjacent non-tumor colorectal tissues using qRT-PCR.
