# Supplementary material for: Antisense lncRNA LDLRAD4-AS1 promotes metastasis by decreasing the expression of LDLRAD4 and predicts a poor prognosis in colorectal cancer
Source: Cell Death Dis. 2020 Feb 28;11(2):155. doi: 10.1038/s41419-020-2338-y (PMC7048743; doi:10.1038/s41419-020-2338-y)
Supplement: Supplementary file 2 — Supplementary Table 1 [file 41419_2020_2338_MOESM2_ESM.docx]

| **Supplementary Table 1. Primers used for PCR validation** | |
| --- | --- |
| Gene | Forward and Reverse primer |
| LDLRAD4 | F: 5' ATGCTTGTATCTTGGTTCG 3' |
|  | R: 5' GATGATGATGATTTGGGCG 3' |
| LDLRAD4-AS1 | F: 5' GAATCTCAGCCTCCCTCTC 3' |
|  | R: 5' TCGTGCTCACTCACTCTCC 3' |
| RP11-701H16.4 | F: 5' GCTTGGTGTGTGTTCCTACC 3' |
|  | R: 5' TAGCACGCACAGAAACTCCC 3' |
| RP11-53B2.1 | F: 5' CTGATCTGGTTGCAGGGACT 3' |
|  | R: 5' TGTAGGCAGAAGGGTTTCGT 3' |
| RP11-53B2.3 | F: 5' TCCTCCTCACGCCACTCATA 3' |
|  | R: 5' CTGGATGGATTGCCAGTGGA 3' |
| RP11-53B2.4 | F: 5' CCAGGCGACAACCGTCTCTA 3' |
|  | R: 5' GCTGCCAAGTTTCCTCTGCT 3' |
| RP11-53B2.5 | F: 5' AATCCACATTCGTGTTGTGGC 3' |
|  | R: 5' TGATGTCAGAGCCTCGTTGA 3' |
| RP11-691H4.3 | F: 5' TGGCTGCTGCACATCATACC 3' |
|  | R: 5' TTGTGTAGGACCGTTGGATGG 3' |
| RP11-691H4.4 | F: 5' CCCCTGCCTACCGATCTTCT 3' |
|  | R: 5' CTGTCTCTTGAGTACCGTGGG 3' |
| β-actin | F: 5' CATGTACGTTGCTATCCAGGC 3' |
|  | R: 5' CTCCTTAATGTCACGCACGAT 3' |
