# Supplementary material for: Antisense lncRNA LDLRAD4-AS1 promotes metastasis by decreasing the expression of LDLRAD4 and predicts a poor prognosis in colorectal cancer
Source: Cell Death Dis. 2020 Feb 28;11(2):155. doi: 10.1038/s41419-020-2338-y (PMC7048743; doi:10.1038/s41419-020-2338-y)
Supplement: Supplementary file 3 — Supplementary Table 2 [file 41419_2020_2338_MOESM3_ESM.docx]

| **Supplementary Table 2. Univariate survival analyses of patients with LDLRAD4 low and high expression** | | | | | | | | |
| --- | --- | --- | --- | --- | --- | --- | --- | --- |
| Variable | No. | 5-year DFS | 5-year CSS | DFS | |  | CSS | |
|  |  |  |  | Log rank χ^2^ | P-value |  | Log rank χ^2^ | P-value |
| LDLRAD4 |  |  |  | 4.527 | 0.033 |  | 5.207 | 0.022 |
| Low | 221 | 59.7% | 65.6% |  |  |  |  |  |
| High | 55 | 75.9% | 83.1% |  |  |  |  |  |
| Gender |  |  |  | 1.406 | 0.236 |  | 0.660 | 0.417 |
| Male | 166 | 60.8% | 68.1% |  |  |  |  |  |
| Female | 110 | 66.3% | 70.9% |  |  |  |  |  |
| Age at diagnosis (yr) |  |  |  | 0.289 | 0.591 |  | 0.820 | 0.365 |
| <60 | 170 | 64.3% | 71.5% |  |  |  |  |  |
| ≥60 | 106 | 60.7% | 65.5% |  |  |  |  |  |
| TNM stage |  |  |  | 183.375 | <0.001 |  | 141.351 | <0.001 |
| I | 21 | 100% | 100% |  |  |  |  |  |
| II | 81 | 86.9% | 88.9% |  |  |  |  |  |
| III | 132 | 60.7% | 68.7% |  |  |  |  |  |
| IV | 42 | 7.8% | 21.5% |  |  |  |  |  |
| T stage |  |  |  | 23.236 | <0.001 |  | 22.094 | <0.001 |
| T2 | 43 | 89.4% | 93.9% |  |  |  |  |  |
| T3 | 54 | 75.3% | 80.6% |  |  |  |  |  |
| T4 | 179 | 53.0% | 61.2% |  |  |  |  |  |
| N stage |  |  |  | 34.425 | <0.001 |  | 27.129 | <0.001 |
| N0 | 120 | 81.9% | 83.8% |  |  |  |  |  |
| N1 | 83 | 54.7% | 63.6% |  |  |  |  |  |
| N2 | 73 | 41.0% | 50.2% |  |  |  |  |  |
| M stage |  |  |  | 167.707 | <0.001 |  | 128.703 | <0.001 |
| M0 | 234 | 73.3% | 78.6% |  |  |  |  |  |
| M1 | 42 | 7.8% | 21.5% |  |  |  |  |  |
| Tumor location |  |  |  | 1.502 | 0.220 |  | 3.567 | 0.059 |
| Colon | 129 | 60.6% | 64.1% |  |  |  |  |  |
| Rectum | 147 | 65.0% | 73.1% |  |  |  |  |  |
| Grade |  |  |  | 0.638 | 0.544 |  | 1.996 | 0.369 |
| Well/ Moderate | 220 | 63.7% | 87.1% |  |  |  |  |  |
| Poor/ Undifferentiated | 56 | 59.8% | 70.4% |  |  |  |  |  |
| Histological type |  |  |  | 0.090 | 0.764 |  | 0.078 | 0.780 |
| Adenocarcinoma | 261 | 62.9% | 68.6% |  |  |  |  |  |
| Mucinous adenocarcinoma | 15 | 65.0% | 79.4% |  |  |  |  |  |
| LNH |  |  |  | 4.369 | 0.037 |  | 3.837 | 0.049 |
| <12 | 81 | 53.2% | 62.7% |  |  |  |  |  |
| ≥12 | 195 | 67.0% | 71.9% |  |  |  |  |  |
| Perineural invasion |  |  |  | 7.278 | 0.007 |  | 4.113 | 0.043 |
| Negative | 231 | 66.9% | 71.5% |  |  |  |  |  |
| Positive | 44 | 44.5% | 56.4% |  |  |  |  |  |
| Vascular invasion |  |  |  | 16.852 | <0.001 |  | 17.294 | <0.001 |
| Negative | 184 | 71.3% | 76.2% |  |  |  |  |  |
| Positive | 88 | 45.4% | 54.3% |  |  |  |  |  |
| Unknown | 4 | 75.0% | 75.0% |  |  |  |  |  |
| Pre-treatment CEA |  |  |  | 27.115 | <0.001 |  | 18.209 | <0.001 |
| Negative | 170 | 74.5% | 78.0% |  |  |  |  |  |
| Positive | 95 | 43.4% | 54.8% |  |  |  |  |  |
| Unknown | 11 | 50.0% | 50.0% |  |  |  |  |  |
| Adjuvant Chemotherapy |  |  |  | 120.122 | <0.001 |  | 120.149 | <0.001 |
| No | 49 | 81.0% | 82.6% |  |  |  |  |  |
| Yes | 189 | 68.6% | 75.3% |  |  |  |  |  |
| Unknown | 38 | 11.7% | 18.4% |  |  |  |  |  |
| MSI status/MMR status |  |  |  | 0.070 | 0.791 |  | 0.124 | 0.725 |
| MSS/MMR-proficient | 176 | 63.0% | 69.8% |  |  |  |  |  |
| MSI/MMR-deficient | 100 | 62.8% | 68.0% |  |  |  |  |  |
| DFS = disease-free survival, CSS = cancer-specific survival, LNH = number of lymph nodes harvested, CEA = carcinoembryonic antigen, MSI = microsatellite instability, MSS = microsatellite stability, MMR = mismatch repair | | | | | | | | |
|  | | | | | | | | |
