# Supplementary material for: Antisense lncRNA LDLRAD4-AS1 promotes metastasis by decreasing the expression of LDLRAD4 and predicts a poor prognosis in colorectal cancer
Source: Cell Death Dis. 2020 Feb 28;11(2):155. doi: 10.1038/s41419-020-2338-y (PMC7048743; doi:10.1038/s41419-020-2338-y)
Supplement: Supplementary file 4 — Supplementary Table 3 [file 41419_2020_2338_MOESM4_ESM.docx]

| **Supplementary Table 3. Multivariate survival analyses of patients with LDLRAD4 low and high expression** | | | | | | | |
| --- | --- | --- | --- | --- | --- | --- | --- |
| Variable | DFS | | |  | CSS | | |
|  | HR | 95% CI | P-value |  | HR | 95% CI | P-value |
| LDLRAD4 |  |  | 0.048 |  |  |  | 0.035 |
| Low | 1 | reference |  |  | 1 | reference |  |
| High | 0.664 | 0.363-0.912 |  |  | 0.597 | 0.292-0.886 |  |
| TNM stage |  |  | <0.001 |  |  |  | <0.001 |
| IV | 1 | reference |  |  | 1 | reference |  |
| III | 0.298 | 0.130-0.682 | 0.004 |  | 0.508 | 0.211-1.223 | 0.131 |
| II | 0.049 | 0.017-0.143 | <0.001 |  | 0.054 | 0.016-0.182 | <0.001 |
| I | -- | -- | 0.947 |  | -- | -- | 0.954 |
| T stage |  |  | 0.046 |  |  |  | 0.045 |
| T2 | 1 | reference |  |  | 1 | reference |  |
| T3 | 3.233 | 1.001-10.448 | 0.050 |  | 5.866 | 1.213-28.362 | 0.028 |
| T4 | 3.634 | 1.273-10.376 | 0.016 |  | 6.494 | 1.489-28.320 | 0.013 |
| N stage |  |  | 0.605 |  |  |  | 0.329 |
| N0 | 1 | reference |  |  | 1 | reference |  |
| N1 | 1.447 | 0.685-3.057 | 0.333 |  | 1.749 | 0.874-3.498 | 0.136 |
| N2 | 1.027 | 0.627-1.683 | 0.916 |  | 2.056 | 0.979-4.317 | 0.271 |
| M stage |  |  | <0.001 |  |  |  | 0.009 |
| M0 | 1 | reference |  |  | 1 | reference |  |
| M1 | 9.743 | 6.313-15.036 |  |  | 3.094 | 1.323-7.236 |  |
| LNH |  |  | 0.172 |  |  |  | 0.154 |
| <12 | 1 | reference |  |  | 1 | reference |  |
| ≥12 | 0.742 | 0.484-1.138 |  |  | 0.708 | 0.440-1.138 |  |
| Perineural invasion |  |  | 0.257 |  |  |  | 0.260 |
| Negative | 1 | reference |  |  | 1 | reference |  |
| Positive | 1.320 | 0.817-2.133 |  |  | 1.363 | 0.795-2.335 |  |
| Vascular invasion |  |  | 0.685 |  |  |  | 0.900 |
| Negative | 1 | reference |  |  | 1 | reference |  |
| Positive | 0.794 | 0.106-5.943 |  |  | 0.778 | 0.104-5.835 |  |
| Pre-treatment CEA |  |  | 0.309 |  |  |  | 0.159 |
| Negative | 1 | reference |  |  | 1 | reference |  |
| Positive | 0.513 | 0.197-1.334 |  |  | 0.386 | 0.145-1.023 |  |
| Adjuvant Chemotherapy |  |  | 0.089 |  |  |  | 0.027 |
| No | 1 | reference |  |  | 1 | reference |  |
| Yes | 2.202 | 0.949-5.108 |  |  | 2.548 | 1.111-5.842 |  |
| DFS = disease-free survival, CSS = cancer-specific survival, LNH = number of lymph nodes harvested, CEA = carcinoembryonic antigen, CI = confidence interval, HR = hazard rate | | | | | | | |
|  | | | | | | | |
