# Supplementary figures and images for: Antisense lncRNA LDLRAD4-AS1 promotes metastasis by decreasing the expression of LDLRAD4 and predicts a poor prognosis in colorectal cancer
Source: Cell Death Dis. 2020 Feb 28;11(2):155. doi: 10.1038/s41419-020-2338-y (PMC7048743; doi:10.1038/s41419-020-2338-y)

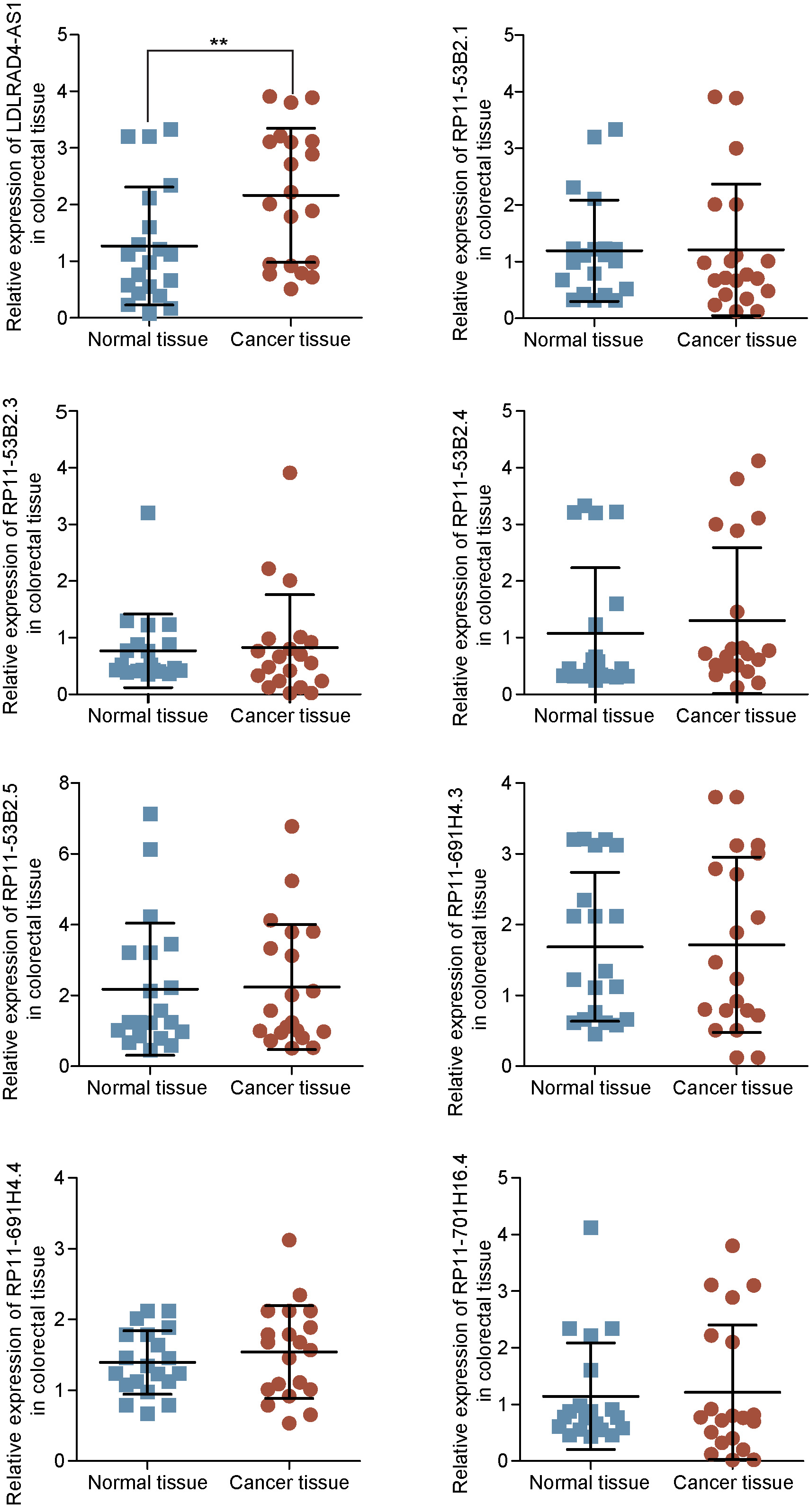

Supplement: Supplementary file 5 — Supplementary Figure 1 [file 41419_2020_2338_MOESM5_ESM.png]
